# Combined Effects of Scarification, Phytohormones, Stratification, and Soil Type on the Germination and/or Seedling Performance of Three Tamaulipan Thornscrub Forest Species

**DOI:** 10.3390/plants11202687

**Published:** 2022-10-12

**Authors:** Paula Luera, Christopher A. Gabler

**Affiliations:** 1 School of Earth, Environmental, and Marine Sciences, University of Texas Rio Grande Valley, 1 W University Blvd, Brownsville, TX 78520, USA; 2 Department of Biology, University of Texas Rio Grande Valley, 1201 W University Dr, Edinburg, TX 78539, USA

**Keywords:** reforestation, germination, propagation, phytohormones, scarification, gibberellic acid, indole-3-butyric acid, Fabaceae, Boraginaceae, Rutaceae

## Abstract

Tamaulipan thornforests in south Texas and northeast Mexico are a conservation hotspot. Shortages of native seedlings limit regional restoration and are largely driven by knowledge gaps regarding propagation of the 75+ thornforest species planted during restorations. We previously investigated three thornforest species with low or inconsistent germination or seedling survival: *Ebenopsis ebano* (Fabaceae), *Cordia boissieri* (Boraginaceae), and *Zanthoxylum fagara* (Rutaceae), and identified the types and dosages of chemical seed treatments that maximized germination. However, chemical treatments were performed in isolation and combinational treatments may be required to break dormancy or maximize germination. This study builds on prior work by investigating the effects of all possible combinations of sulfuric acid (SA), gibberellic acid (GA), and indole-3-butyric acid (IBA) treatments on germination of the same focal species, and further quantified the combined effects of five chemical treatments, three stratification treatments, and six soil mixture types on the germination and seedling performance of the focal species. *Ebenopsis ebano* germination peaked with SA and was not improved with additional chemical treatments. *Cordia boissieri* germination was highest with GA only in our indoor experiment but peaked with GA + IBA + SA in our outdoor experiment. *Zanthoxylum fagara* germination was near zero in all treatments. Stratification treatments marginally reduced *E. ebano* germination and reduced *C. boissieri* seedling height. Soil type had significant impacts on *E. ebano* germination and leaf abundance (residual differences up to 40% or 4 leaves, respectively) and influenced some of the effects of chemical treatments. These results enhance our understanding of thornforest seed ecology and best practices for nursery propagation of seedlings.

## 1. Introduction

Deforestation continues and the net annual change in global forest cover remains negative, despite growing reforestation efforts and widespread recognition of the importance of forests for human wellbeing, biodiversity, and overall biosphere health [1,2]. Forests provide a multitude of ecosystem functions and services, including biodiversity support (wildlife habitat), carbon storage, climate regulation, and provisioning of air, water, and food [1,2,3]. However, resources and human support for the protection of intact forest habitats and restoration of lost or degraded habitats are finite, so it has been recommended that we prioritize forests that most effectively mitigate climate change and provide important local socio-ecological functions, e.g., providing food in areas with high food insecurity or wildlife habitat in areas with high biodiversity or conservation value [3].

In the Lower Rio Grande Valley (RGV) of southern Texas and in northeastern Mexico, less than 2% of the historic Tamaulipan (or Mezquital) thornscrub forests (or thornforests) remain [4]. These forests exhibit high biodiversity, with hundreds of plant species supporting an array of migratory and resident birds, insects (especially bees and butterflies), mammals, and reptiles, including many species endemic to the region and threatened at the state or national level [5,6], such as the federally endangered ocelot (*Leopardus pardalis*) [7,8,9,10]. Tamaulipan thornforests also support a critical ecotourism industry valued at 59–300 million USD per year in a region with high poverty and food insecurity [11]. Nevertheless, thornforests are threatened by rapid urbanization and are already heavily fragmented due to over a century of agricultural modification, which has reduced biodiversity and forest cover across the region [5,12,13]. Given its high biodiversity and ecological importance paired with its high risk from human impacts, the Tamaulipan ecoregion has been identified as a conservation hotspot [14]. As a result, restoration of Tamaulipan thornforests are a high priority for various governmental, conservation, and commercial organizations who operate in the region and collaborate to produce and plant native thornforest species on both public and private lands [4,14].

Native plant seedling availability is currently the greatest limiting factor for Tamaulipan thornforest restoration [15]. Current best practices for forest restoration in the Rio Grande Valley (RGV) of south Texas require the planting of seedlings, rather than seeds, and for all seedlings to be grown from locally collected seed to increase genetic diversity in these habitats while promoting locally adapted genotypes [16]. However, native seed availability is also a limiting factor. Native seed is only commercially available in small quantities and for a few of the 75+ species regularly planted for restoration, and wild collection and processing of native seed is both labor-intensive and requires significant expertise and local knowledge. Similarly, propagation of these 75+ native species from seed is also labor- and knowledge-intensive, and germination and/or seedling survival are often low or inconsistent, partly because many knowledge gaps remain about best practices for nursery propagation [15]. Consequently, nursery production of thornforest seedlings is high risk and difficult for commercial growers, which limits the number of qualified growers producing seedlings and creates a major bottleneck in reforestation capacity. Greater understanding of the horticultural techniques required to break seed dormancy, enhance germination, and maximize post-germination and post-transplant survival for Tamaulipan thornforest species is urgently needed [15].

Multiple mechanisms underlie seed dormancy and the requirements to break dormancy can vary widely among species within the same region [17]. Baskin and Baskin [18] identified five classes of seed dormancy: physical, physiological, morphological, morphophysiological, and combinational. Generally, seeds experience a combination of natural processes that can act to break dormancy, including daily and seasonal temperature fluctuations, weathering by wind and water, gut passage, and desiccation [17,19]. In the RGV, the climate is semiarid and borderline tropical-subtropical [5], which promotes considerable variation among thornforest plant species in both the timing of flowering, fruiting, and germination, and in the nature of phenological cues, with rainfall pulses and seasonal shifts in temperature or day length hypothesized to be most common, but the exact nature of phenological and germination triggers are poorly understood for most species [20,21]. Our understanding of the role temperature plays in the seed and seedling behavior of Tamaulipan thornforest species is underdeveloped [21,22,23]. Long-established horticulture practices, such as cold stratification, mimic the seasonal temperature conditions that govern dormancy in many species [24], for example, by exposing seeds to an identified number of cold hours at 4–10 °C via refrigeration [17]. Alternatively, ovens or driers can be used to affect other environmental triggers, such as after ripening, seed desiccation, and exposure to high temperatures [25,26]. Although these methods are not new, their applications to thornscrub species are largely untested, despite demonstrated utility for some species [23]. Understanding thermal triggers of germination will help us better understand thornscrub ecology and community dynamics and would be of significant practical use in breaking seed dormancy for propagation.

In our previous study, Luera et al. [15] performed a series of scarification and phytohormone trials to investigate the factors governing seed dormancy in three focal thornforest species: *Ebenopsis ebano* (Berl.) Barneby & Grimes (Texas ebony), *Cordia boissieri* A. DC. (Mexican olive), and *Zanthoxylum fagara* (L.) Sarg. (colima). Seed scarification with sulfuric acid increased *E. ebano* germination, suggesting physical dormancy, whereas seed treatment with the phytohormone gibberellic acid increased *C. boissieri* germination, suggesting physiological dormancy [15]. However, scarification and phytohormone treatments were tested in isolation, so we could not rule out combinational (physical plus physiological) dormancy, and, although we investigated the effects of temperature and soil type on seedling growth and root morphology, we did not study their effects on germination. This study builds on our prior work in three important ways. (1) We included seed treatments that combined scarification and different phytohormones. (2) We investigated the effects of temperature and soil type—in conjunction with scarification and phytohormones—on germination. (3) We tested germination outdoors in soil, which better reflects the realities of thornforest seedling production.

This study focused on the same three focal Tamaulipan thornforest species as Luera et al. [15] and employed two factorial experiments for each species. The first experiment quantified the effects of the optimal dosages of sulfuric, gibberellic, and indole-3-butyric acids identified by Luera et al. [15] on germination when applied to seeds alone and in all possible combinations. The second experiment quantified the effects of a subset of five chemical seed treatments, six soil mixture types, and three stratification treatments on the germination of our three focal species and on the seedling performance of *E. ebano*, using a full factorial design. Many knowledge gaps remain regarding the propagation of Tamaulipan thornforest plant species, which, when filled, should reduce risk and increase profitability for commercial growers seeking to produce native thornforest species [15]. In turn, commercial viability of thornforest seedling production should promote increased availability of thornforest seedlings, which currently limits restoration in the region. This study increased our understanding of thornforest seed dormancy and further elucidated best practices for enhancing germination and seedling performance in a nursery setting.

## 2. Results

### 2.1. Ebenopsis Ebano Small Factorial Experiment

**Figure 1:**
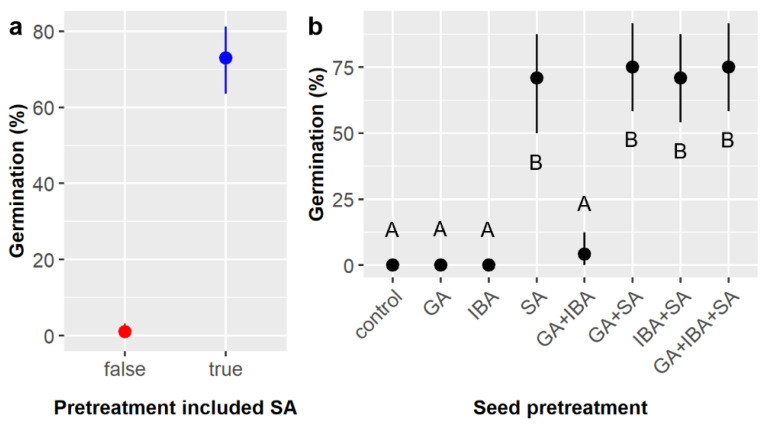
Average germination likelihood of *Ebenopsis ebano* seeds with 95% confidence intervals broken down by (**a**) whether sulfuric acid (SA) scarification was included as part of the seed pretreatment (to show the main effect of SA; red dot = SA not included, blue dot = SA included); and (**b**) pretreatments imposed on *Ebenopsis ebano* seeds, which included gibberellic acid (GA), indole-3-butyric acid (IBA), sulfuric acid (SA), and all combinations thereof. Capital letters denote the results of least squares means post-hoc tests; groups within a panel that share a letter were not significantly different.

Consistent with prior results [15], treatment of *E. ebano* seeds with sulfuric acid (SA) was required for germination. SA significantly increased the likelihood of germination from 1.0% to 72.9% overall (Table 1, Figure 1a). However, no other chemical treatments or interactions among treatments significantly influenced germination likelihood (Table 1), and post-hoc tests showed that there were no significant differences in germination likelihood among the treatment combinations that included SA, namely SA, GA + SA, IBA + SA, and GA + IBA + SA, which ranged from 70.8% to 75.0% germination (Figure 1b).

**Figure 2:**
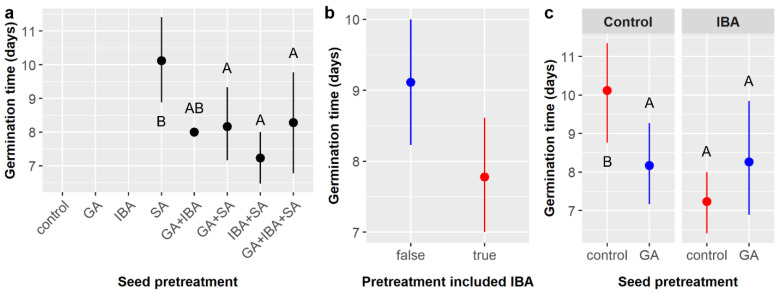
Average time to germination of *Ebenopsis ebano* seeds with 95% confidence intervals broken down by (**a**) all pretreatments imposed, which included gibberellic acid (GA), indole-3-butyric acid (IBA), sulfuric acid (SA), and all combinations thereof; (**b**) whether IBA was included in the seed pretreatment (to show the main effect of IBA; blue dot = IBA not included, red dot = IBA included); and (**c**) GA and IBA treatments (to show the GA × IBA interactive effect; red dots = GA not included, blue dots = GA included). Capital letters denote the results of least squares means post-hoc tests; groups within a panel that share a letter were not significantly different.

Time to germination averaged 8.4 ± 2.8 days across all treatments (Figure 2a) and was significantly influenced by IBA and the GA × IBA interaction only (Table 2). Germination was 1.3 days faster with IBA treatment overall (7.8 vs. 9.1 days) (Figure 2b), but the effect of IBA appears to be dependent on the GA treatment. Without GA, IBA treatment reduced germination time from 10.1 to 7.2 days, but germination time with GA was similar with or without IBA (8.3 days for GA + IBA, 8.2 days for GA) (Figure 2c). Since over 98% of germinated *E. ebano* seeds were treated with SA, it is most appropriate to interpret the effects of GA and IBA on germination timing as being those observed in conjunction with SA.

### 2.2. Ebenopsis Ebano Large Factorial Experiment

**Figure 3:**
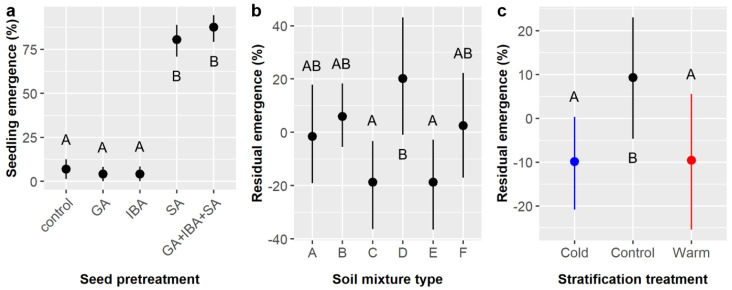
Average likelihoods of *Ebenopsis ebano* seedling emergence with 95% confidence intervals broken down by (**a**) chemical seed pretreatment, which included controls, gibberellic acid (GA), indole-3-butyric acid (IBA), sulfuric acid (SA), and GA + IBA + SA; (**b**) soil mixture type; and (**c**) stratification treatment (blue dot = cold, black dot = control, red dot = warm). Panels (**b**,**c**) show the residual values and their 95% confidence intervals for emergence likelihoods. Capital letters denote the results of least squares means post hoc tests; groups within a panel that share a letter were not significantly different.

Across all treatments, 36.7% of *E. ebano* seeds germinated and emerged from the soil surface. Chemical seed pretreatment, soil mixture type, and the interaction of chemical pretreatment and stratification significantly influenced the likelihood of seedling emergence (Table 3). Again, emergence was highest among pretreatments that included SA (SA = 80.6%, GA + IBA + SA = 87.5%) and dramatically lower without SA (GA = 4.2%, IBA = 4.2%, control = 6.9%) (Figure 3a). The highest emergence occurred in soil type D (45%), which was significantly higher than in soils C (30%) and E (30%) (Figure 3b). Stratification had a marginal effect on emergence (Table 3), but post-hoc tests suggested that emergence was higher in the control (41.7%) than in either the warm or cold stratification treatments, which both averaged 34.2% emergence (Figure 3c). The significant pretreatment × stratification interaction (Table 3) arose because the observed pretreatment effects varied among the stratification treatments. Specifically, emergence was low among the non-SA pretreatments (GA, IBA, and control) in the warm and control stratification treatments (4.2–12.5%) but was zero for these three pretreatments in the cold stratification treatment.

Several response variables could only be quantified if emergence occurred, namely time to emergence, seedling survival, height, and leaf count. For these variables, the overwhelming majority of emergence occurred in categories that included SA, so we excluded any pretreatment categories that lacked SA from our analyses (i.e., we kept only SA and GA + IBA + SA). Including all pretreatment categories resulted in strongly unbalanced sample sizes and structural zeroes for some treatment combinations and, practically, SA treatment has now been established as a standard practice for *E. ebano* propagation from seed. Thus, for this group of response variables, it is both statistically and practically appropriate to perform analyses that include only the SA and GA + IBA + SA pretreatments.

**Figure 4:**
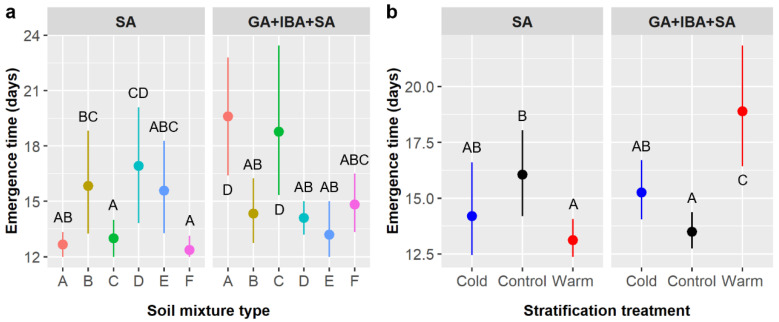
Average time to *Ebenopsis ebano* seedling emergence with 95% confidence intervals broken down by (**a**) soil mixture type and the two chemical pretreatments that included SA (dot colors correspond to the six soil mixture types and are included to improve readability); and (**b**) stratification and pretreatment (blue dots = cold, black dots = control, red dots = warm). Capital letters denote the results of least squares means post-hoc tests; groups within a panel that share a letter were not significantly different. Values shown include observations only from pretreatment groups that included SA.

Time to emergence averaged 15.2 ± 4.4 days and was significantly influenced by pretreatment and the interactions of pretreatment × soil, pretreatment × stratification, and soil × stratification (Table 4). Emergence time was 14.6 days with SA only and 15.7 days when SA was combined with GA and IBA. However, the effect of pretreatment also depended on soil type (pretreatment × soil interaction) and stratification (pretreatment × stratification interaction). In the SA treatment compared to the GA + IBA + SA treatment, emergence was faster in soils A (12.7 vs. 19.6 days) and C (13.0 vs. 18.8 days) but slower in soil D (16.9 vs. 14.1 days) (Figure 4a), and emergence was faster with warm stratification (13.1 vs. 18.9 days) but slower in controls with no stratification (16.1 vs. 13.5 days) (Figure 4b). The effect of stratification on emergence time also depended on soil type (soil × stratification interaction); most treatments averaged 15 or fewer days to emergence, except controls in soil D (18.1 days) and warm stratification in soils A (23.3 days) and C (20.0 days), which were significantly slower than most other treatment combinations (not shown).

Survival of *E. ebano* seedlings was 91.7% across all treatments. None of the factors tested significantly influenced survival, but soil had a marginal effect on survival (Table 5). Survival was highest in soils C, D, and F (100%, 95.2%, and 100%, respectively) and lowest in soils A, B, and E (85.0%, 87.5%, and 83.3%, respectively), but both our post-hoc tests and GLM suggested that none of these differences were significant (not shown).

**Figure 5:**
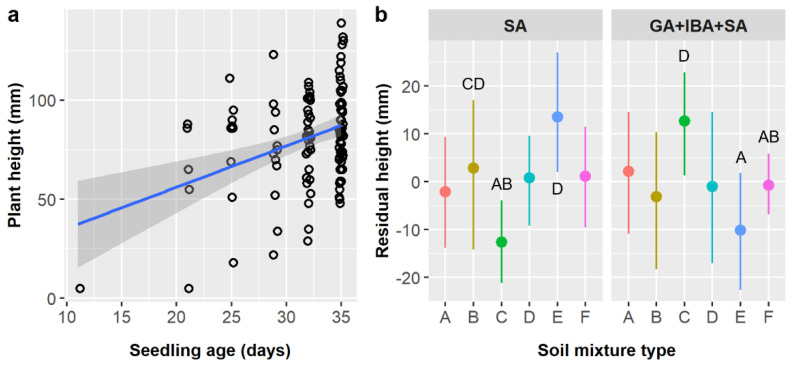
(**a**) Scatterplot with trendline showing the positive linear relationships between *Ebenopsis ebano* seedling age and plant height; each dot represents one plant. (**b**) Average residual plant height with 95% confidence intervals broken down by soil mixture type and the two pretreatments that included SA (dot colors correspond to the six soil mixture types and are included to improve readability). Capital letters denote the results of least squares means post-hoc tests; all groups without labels are in the ‘ABCD’ post-hoc group (labels omitted for clarity); groups within a panel that share a letter were not significantly different. Values shown include observations only from pretreatment groups that included SA.

We considered seedling age in our analyses of *E. ebano* seedling height and leaf abundance because seedlings emerged at different times and age influences size. Seedling height averaged 79.5 ± 26.5 mm overall and was significantly influenced by seedling age and the interaction of pretreatment and soil (Table 6). The age × stratification and soil × stratification interactions also had marginal effects on height. Height increased by 3.18 mm per day (Figure 5a). As before, the effect of pretreatment depended on soil type (pretreatment × soil interaction); residual seedling heights were 25.3 mm greater for soil C and 23.6 mm lower for soil E in the GA + IBA + SA treatment than in the SA only treatment, but height was not significantly different between treatments for other soil types (Figure 5b). Similarly, the effect of stratification varied among soil types (stratification × soil interaction), with residual heights 25.4 and 24.8 mm greater in cold than in warm treatments for soils C and E, respectively, and 23.3 mm greater in warm than in control treatments for soil B; however, these differences were marginal in our ANCOVA and only the differences for soils B and C were significant in our post-hoc tests. The relationship between seedling age and height was weaker in the control stratification treatment (m = 0.005) than in the cold (m = 0.293) or warm (m = 0.279) treatments (age × stratification interaction), but these differences were only marginal in our ANCOVA (Table 6). 

**Figure 6:**
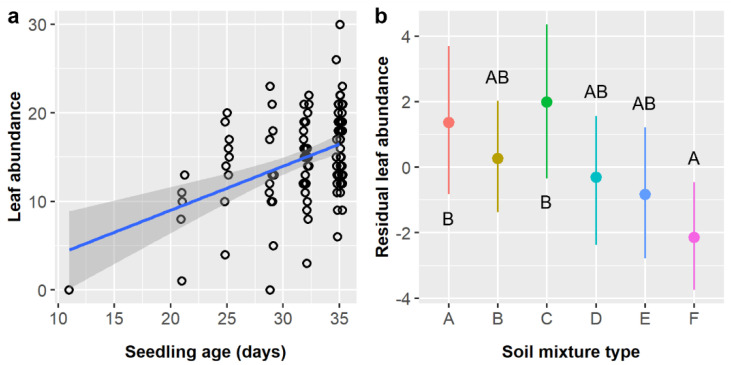
(**a**) Scatterplot with trendline showing the positive linear relationships between *Ebenopsis ebano* seedling age and leaf abundance; each dot represents one plant. (**b**) Average residual leaf abundance with 95% confidence intervals broken down by soil type (dot colors correspond to the six soil mixture types as above to aid comparisons). Capital letters denote the results of least squares means post-hoc tests; groups within a panel that share a letter were not significantly different. Values shown include observations only from pretreatment groups that included SA.

*Ebenopsis ebano* leaf abundance averaged 14.6 ± 5.5 leaves and was influenced by seedling age and soil type (Table 7). Seedlings gained 0.61 leaves per day of growth (Figure 6a) and had significantly more leaves when grown in soil types A or C (15.5 or 16.3 leaves, respectively) than in soil F (13.4 leaves), while soils B, D, and E produce intermediate leaf counts (Figure 6b).

### 2.3. Cordia Boissieri Small Factorial Experiment

**Figure 7:**
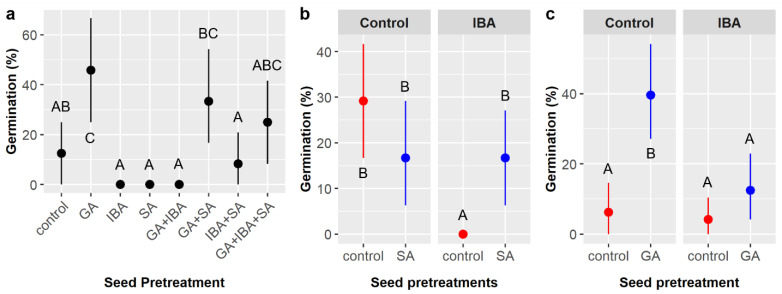
Average germination likelihood of *Cordia boissieri* seeds with 95% confidence intervals broken down by (**a**) seed pretreatments, which included gibberellic acid (GA), indole-3-butyric acid (IBA), sulfuric acid (SA), and all combinations thereof; (**b**) IBA and SA treatments (red dots = SA not included, blue dots = SA included); and (**c**) GA and IBA treatments (red dots = GA not included, blue dots = GA included). Capital letters denote the results of least squares means post-hoc tests; groups within a panel that share a letter were not significantly different.

Overall, 15.6% of *C. boissieri* seeds germinated in the smaller factorial experiment. Gibberellic acid (GA), indole-3-butyric acid (IBA), and the interaction of IBA and sulfuric acid (SA) had significant effects on germination likelihood, while the GA × IBA interaction had a marginal effect (Table 8). Figure 7a illustrates germination likelihood across all seed pretreatment combinations. Overall, treatment with GA increased germination by over 20%, a five-fold increase (26.0% with GA vs. 5.2% without GA), but IBA treatment decreased germination by about 15% (8.3% with IBA vs. 22.9% without IBA), a nearly threefold decrease. However, SA treatment appears to partially negate the effect of IBA (IBA × SA interaction). With SA, germination was identical with or without IBA (16.7%), but, without SA, zero seeds germinated when treated with IBA (0%) and 29.2% germinated without IBA (Figure 7b). The marginal GA × IBA interaction suggests combining GA and IBA may have had a non-additive effect; specifically, germination in GA treatments without IBA (39.6%) was 27.1% higher than in GA treatments with IBA (12.5%), which was a greater difference than the 15% increase expected based on the main effect of IBA (Figure 7c).

Time to germination for *C. boissieri* averaged 10.0 ± 3.7 days and was significantly affected by SA treatment only (one-way ANOVA, *F*_1,28_ = 5.16, *p* = 0.0310). Germination time was 2.9 days slower for seeds treated with SA (11.3 days) compared to those without SA (8.4 days). More complex models with terms for GA, IBA, SA, and their interactions also suggested that only SA was significant, but the overall models were not significant, and stepwise model building and pruning functions in R both returned models with SA as the sole term.

### 2.4. Cordia Boissieri Large Factorial Experiment

**Figure 8:**
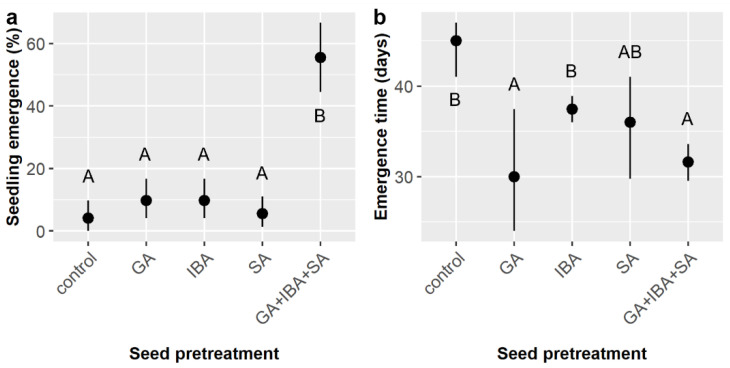
(**a**) Average likelihood of seedling emergence and (**b**) average time to emergence for *Cordia boissieri* seeds with 95% confidence intervals broken down by seed pretreatment, which included controls, gibberellic acid (GA), indole-3-butyric acid (IBA), sulfuric acid (SA), and GA + IBA + SA treatments. Capital letters denote the results of least squares means post-hoc tests; groups within a panel that share a letter were not significantly different.

Similar to the smaller experiment, seedling emergence was observed for 17.0% of the *C. boissieri* seeds planted, overall. Chemical seed pretreatment and the interaction of pretreatment and soil type significantly influenced emergence likelihood (Table 9). However, unlike the smaller experiment, emergence did not peak with GA treatment alone (9.7%) and was instead enhanced and maximized when pretreatments were combined (55.6% in the GA + IBA + SA treatment) (Figure 8a). Emergence in different soil treatments ranged from 41.7 to 66.7% within the GA + IBA + SA treatment, but none were statistically different; the pretreatment × soil interaction arose from variability within soil types across the control, GA, IBA, and SA chemical treatments, which ranged from zero (0%) to 25.0% without any apparent pattern (not shown).

Time to emergence averaged 33.1 ± 7.5 days, which was slightly more than double the germination time observed in the smaller factorial experiment. Pretreatment was the only factor to influence emergence time (Table 10). GA and GA + IBA + SA had the shortest emergence times (30.0 and 31.6 days, respectively), which were significantly faster than the IBA and control treatments (37.4 and 45.0 days, respectively), whereas SA was intermediate (36.0 days) and highly variable (Figure 8b).

*Cordia boissieri* seedling survival averaged 93.4% across all treatments but was not significantly influenced by any of our experimental treatments (Table 11).

**Figure 9:**
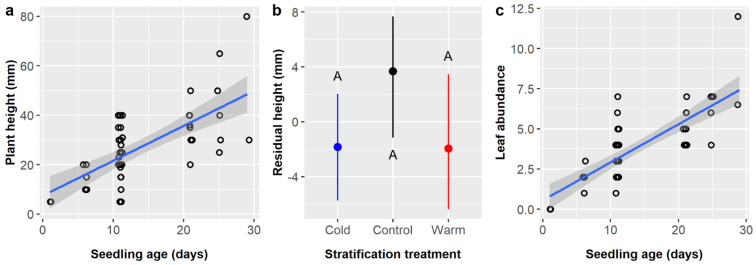
Scatterplots with trendlines showing the positive linear relationships between *Cordia boissieri* seedling age and (**a**) plant height and (**c**) leaf abundance; each dot represents one plant. (**b**) Average residual plant height with 95% confidence intervals broken down by stratification treatment (blue dot = cold, black dot = control, red dot = warm). Capital letters denote the results of least squares means post-hoc tests; groups within a panel that share a letter were not significantly different.

*Cordia boissieri* seedling height averaged 25.9 ± 15.1 mm and depended upon seedling age and stratification, while pretreatment had a marginal effect (Table 12). Seedling height increased by 1.23 mm per day of growth (Figure 9a). Residual seedling height was ca. 5.5 mm higher in controls (3.67 mm) than in either cold (−1.84 mm) or warm (−1.94 mm) stratification treatments, but post-hoc tests did not detect any significant differences between stratification treatments (Figure 9b). Residual height values were highest in the control (1.75 mm) and GA treatments (2.25 mm), near zero for IBA (0.22 mm) and GA + IBA + SA (−0.09 mm), and lowest in SA (−5.17 mm), but post-hoc tests detected no significant differences between pretreatment groups.

Finally, leaf abundance averaged 3.65 ± 2.24 leaves and was significantly influenced by seedling age only (Table 13). *Cordia boissieri* seedlings gained 0.22 leaves per day of growth (Figure 9c).

### 2.5. Zanthoxylum Fagara Small and Large Factorial Experiments

*Zanthoxylum fagara* exhibited zero germination across all treatments in both factorial experiments. This was likely due to extremely low viability of the seeds tested, but we did not test viability separately via alternative means. The implications of this result are discussed below.

## 3. Discussion

Large-scale reforestation requires large-scale seedling production in many regions, including the Tamaulipan biotic province, where seedling availability is the principal limiting factor [15,27]. Enhanced production of native plant seedlings in south Texas could both increase the acreage of thornforests restored each year and promote regional economic growth if the best practices for successful nursery propagation are identified and made readily accessible to current and future growers. Luera et al. [15] previously studied the effects of single-chemical seed treatments on the germination and early growth of Tamaulipan thornforest tree species. They tested different dosages of GA, IBA, and SA but did not test combinational treatments, which were a key focus of the current study and may be required for species that exhibit combinational dormancy. We used the optimal dosages for single-chemical treatments identified by Luera at al. [15] as the basis for our combinational treatments and included the same single-chemical treatments in the current study to permit direct comparisons. Our results both corroborate prior results and provide new findings that enable additional refinement of current best practices for Tamaulipan thornforest species propagation.

First, this study confirmed that treatment of *E. ebano* seeds with SA for 50 min effectively triggered germination and demonstrated for the first time that neither preliminary stratification treatments nor subsequent treatment of SA-treated seeds with GA and/or IBA further increased germination likelihood beyond the effect of SA (Figure 1). However, subsequent GA and/or IBA treatment did reduce time to germination by ca. 2–3 days (Figure 2), and germination likelihood was ca. 4% higher (not statistically significant) (Figure 1 and Figure 3) with subsequent GA treatment in both our small and large *E. ebano* experiments. These impacts are probably too minor to justify the use (and cost) of GA in practice, and the use of IBA in powder form is even less justified, but this modest increase suggests that *E. ebano* may also exhibit some weak physiological dormancy mechanisms that are relatively easily overcome. Though not directly tested previously, some level of physiological dormancy is suggested by prior thornforest studies that found environmental impacts on *E. ebano* germination [21,22,23]. Although combinational chemical treatments did not significantly enhance germination beyond the previously identified optimal methods [15], there is value in ruling out unnecessary treatments and materials. Fortunately, the best practice identified herein of treating *E. ebano* seeds with SA only is both cost- and labor-efficient at large scales, especially compared to more labor-intensive scarification methods like nicking [28]. Treatment with GA remains worth considering for hard-to-germinate *E. ebano* seeds, and the weak effects of IBA may be related to our use of its powder form, which could be inherently less effective when treating seeds; both merit further evaluation.

Prior studies showed that soil mixture composition had weak but significant effects on *E. ebano* seedling survival and somewhat stronger effects on seedling growth, especially belowground [15]. In our large factorial experiments, we tested germination (i.e., emergence) when seeds were planted outdoors in soil, which is much closer to normal nursery conditions than prior germination tests using incubated petri dishes, and we found that soil impacted germination likelihood (Table 3, Figure 3b) more strongly than it influenced seedling survival or performance (Table 5 and Table 7, Figure 5). Other studies involving *E. ebano* and other thornforest species recognize the importance of edaphic properties to germination and early seedling growth and survival but did not manipulate soil types or soil properties as was done in this study [22,23].

*Ebenopsis ebano* seeds were 15% more likely to germinate and emerge in soil type D than in soils C or E, and residuals analysis suggests even greater variation in emergence (ca. 40%) between these groups that is attributable explicitly to soil type (Figure 3b). We observed only a marginal effect of soil on survival (*p* = 0.076), but the differences between soil types was greater (83–100%) than in Luera et al. [15] (92–100% in unheated treatments). In both studies, the same soil types were used and soil types A, B, and E tended to have lower survival overall than soils C, D, and F. All soil mixture types tested had a low bulk density (0.48–0.89 g/cm^3^ dry weight) [15], which appears unrelated to survival in this study, partly because C and F have the highest and lowest bulk densities, respectively, yet both exhibited 100% seedling survival. This is somewhat surprising because bulk density impacts plant performance and distributions, but all soil types tested had densities below levels associated with suppression of root growth [29]. Survival likelihoods appear to correlate more with water-holding capacity, which we estimated to be highest in soils D and F and lowest in B. This finding agrees with the many studies that have shown drought stress and soil water holding capacity are critical to plant recruitment and regeneration in thornforests and other dryland ecosystems [23,30,31].

Soil had only weak and marginally significant main effects on seedling height in both this study and its predecessor (Table 6), yet, in both, soil type F produced the shortest seedlings while soils B, C, and D produced the tallest [15]. This is somewhat surprising, given the importance of water availability for all plant growth and that the soil mixtures tested varied in their water holding capacity, but these studies were performed in a nursery context with watering regimes designed to keep plants well-watered. Notably, the effect of treating *E. ebano* seeds with GA and IBA after SA treatment depended on soil type (pretreatment × soil interaction, *p* = 0.0172). There was no effect for most soil types, but, in soils C and E, the residual height difference was ca. 2 cm, which is considerable given the average seedling was only 8.0 cm (Figure 5b). The mechanism underlying this interactive effect is unclear, but this pattern may suggest that the powder form of IBA had an impact, but its ability to persist and take effect is governed by soil properties.

Leaf abundance was significantly influenced by soil type in this study and its precursor (Table 6), but which soil types had the most leaves differed between studies [15]. Previously, in unheated controls, soils E and F had the most leaves and soils A and C had the fewest, whereas the opposite was true in this study (Figure 6b). Given that watering and shade protocols were the same and climatic conditions were comparable in this experiment and in Luera et al. [15], these differences in leaf abundance most likely reflect age-related differences in plant growth (and growth strategies) and the fact that seedlings of different ages were more evenly distributed among soil treatments in the prior study. Previously, seedlings were assigned to soil treatments upon germination in petri dishes, but here, seeds were planted and allowed to germinate in assigned soil treatments, which differed in their germination timing in interaction with other factors (Table 4). This resulted in differences in seedling ages between treatment groups, and age was the strongest predictor of leaf abundance in both studies. The current results are more applicable in a practical nursery context, where differences in germination times could overshadow differences in growth rates between soils (depending on the magnitudes of differences and the duration of the growth period).

Prior examination of *E. ebano* performance within these soil types suggested that soil microbial communities may play an important role in seedling growth. This is broadly true across plant communities and may be particularly important in stressful environments [32,33,34]. Soil types D (50% peat moss, 25% perlite, and 25% vermiculite) and E (50% peat moss, 25% perlite, 25% sand) contained no live topsoil, and D is a standard substrate utilized in many commercial nurseries, yet the presence of topsoil was not associated with any clear trends in seedling survival or performance. *Ebenopsis ebano*, like other members of the Fabaceae, are capable of fixing nitrogen through soil microbial interactions, so the use of fertilizer in this study may have masked some microbial impacts on *E. ebano* growth that are more important outside of a nursery context (i.e., post-transplantation). Future studies of microbial impacts on seedling performance and post-transplant establishment are merited across thornforest species, in part because inclusion of topsoil or soil inoculants may have strong effects for some species [34].

Stratification was not tested in the prior study [15], but here it had a marginal main effect on *E. ebano* emergence likelihood, and it had interactive effects on emergence likelihood (Table 3) and timing (Table 4) and marginally on seedling height (Table 6). However, there was no evidence to warrant cold or warm stratification treatment when propagating *E. ebano* from seed; rather, stratification generally had subtle negative effects that varied in intensity among soil and chemical treatments. These findings agree with prior studies that found little effect of temperature on *E. ebano* germination and early growth [22]. However, these findings have larger implications for seed storage, which is an important practical consideration. The lack of significant negative effects of cold stratification on germination likelihood validates the use of cold storage, which can buffer against seed shortages in years with low seed production. However, our cold stratification treatment was only 30 days and not as cold as most cold storage facilities, so due caution is merited when storing *E. ebano* seeds, but our results agree with years of observations by United States Fish and Wildlife Service (USFWS) personnel, who have been using cold storage for *E. ebano* for years and observed no obvious negative effects of the practice [35]. Seed longevity (viability over time) for different thornforest species merits future evaluation, as it has scarcely been quantified, as do tests of whether different storage regimes (e.g., cold vs. dry) can improve longevity.

Our lab tests (small factorial experiment) with *C. boissieri* confirmed prior findings by Luera et al. [15] that the main effects of GA treatment had a strong positive effect and SA had no effect on germination likelihood (Table 8, Figure 6). However, the prior study found IBA had no effect, but we found that the main effect of IBA significantly decreased germination likelihood. The positive effects of GA on germination are well-established for many species [36,37,38], whereas evidence for germination benefits from IBA treatment are much more limited [39], but a negative effect of IBA was unexpected. Unlike *E. ebano*, interactions among chemical treatments had clear and relatively strong effects on germination likelihood. The positive effect of GA appears to be mediated by IBA (Figure 6a), and the negative effect of IBA appears to be mediated by SA (Figure 6b). There was not a significant GA × SA interaction, but germination was significantly (13%) lower with GA + SA treatment than GA alone, suggesting that sulfuric acid may have damaged the embryo, or that residual SA in the pericarp reduced the efficacy of gibberellic acid.

Importantly, our outdoor tests (large factorial experiment) disagreed with the current and prior lab tests [15] in a critical way: under realistic nursery conditions, treatment with GA alone did not significantly increase germination likelihood relative to other single chemical treatments or controls (Table 9, Figure 7a). However, the combined GA + IBA + SA treatment increased germination likelihood over five-fold relative to single-chemical seed treatments and the control. The exact mechanisms underlying these differing results are uncertain, but we hypothesize that both endocarp permeability and the leaching of phytohormones out of containers are important factors. In lab tests, seeds were in closed petri dishes and only ever rinsed according to surface sterilization protocols to minimize molding, as described in Luera et al. [15], whereas, in nursery tests, seeds were in well-drained containers that were watered regularly and exposed to rain. Thus, any residual GA, IBA, or SA on or in the porous endocarp of *C. boissieri* likely leached away much faster in nursery tests compared to lab tests, thereby reducing the total amount of aqueous GA able to penetrate the endocarp and diffuse into embryonic tissue. Meanwhile, the scarifying effect of SA treatment should render the endocarp more permeable to GA, more porous overall (which would increase the endocarp surface area and allow it to absorb and hold more aqueous GA), or both, thereby potentially offsetting the reduction in GA reaching the *C. boissieri* embryo due to increased leaching. Therefore, in nursery conditions, GA would only be effective if seeds were first treated with SA.

There is evidence to support this hypothesized mechanism, yet additional tests to explain the discrepancy in the effects of GA on *C. boissieri* germination between nursery and lab experiments are merited. First, although SA had no effect on *C. boissieri* germination in this lab experiment or prior studies, Luera et al. [15] showed that physically cracking *C. boissieri* seeds increased germination likelihood from 9% to 40%. Together, these findings suggest that the permeability of the *C. boissieri* endocarp influences germination, but that SA does not effectively render the endocarp permeable to water. Rather, heating and drying of *C. boissieri* seeds resulted in the fissuring of the endocarp, which is far more likely to occur outdoors where temperatures and soil moisture levels fluctuate much more frequently and strongly than in an incubator. This would also explain why seeds treated with GA after being treated with SA were not any more likely to germinate than seeds treated with only GA in the current laboratory experiment (Figure 6a), but they were more likely to germinate in the outdoor experiment (Figure 7a). SA treatment likely both increases the porosity of *C. boissieri* endocarps and facilitates thermal/desiccative cracking. A prior study found that warm stratification increased *C. boissieri* germination [40], which concurs with this notion of heating to induce fissuring of the endocarp, but more directly disagrees with our finding that warm stratification had a negative effect on *C. boissieri* germination.

The same leaching effect may also explain why IBA significantly reduced *C. boissieri* germination likelihood in our indoor experiment (Table 8, Figure 6) but had no effect in our outdoor experiment (Table 9, Figure 7). IBA did not reduce germination in a previous indoor experiment with *C. boissieri*, but its germination was so low overall that such an effect was likely undetectable, and the same study found that IBA increased the rate of seed molding, likely due to the anti-caking agent present in the powder form of IBA utilized [15]. If IBA presence was deleterious, then the higher leaching rates in the outdoor experiment could have reduced IBA concentrations and its overall effect. Alternatively, if IBA reduced germination by promoting molding, the regular cycle of soils drying out between watering in the outdoor experiment may have mitigated the negative effects of mold proliferation.

The mechanism behind the mediating effect of SA on the negative effect of IBA (Figure 6b) is unknown. It is conceivable that residual SA left in the endocarp after scarification created a high-pH barrier that acted to degrade IBA diffusing toward the embryo, impeded mold penetration, or both. If residual SA degraded IBA entering the seed, it could have had a similar effect on GA, and this may explain why germination was lower in the GA + SA treatment compared to the GA only treatment in the lab experiment with less leaching (Figure 6a) but higher in the GA + IBA + SA treatment compared to GA only in the outdoor experiment with more leaching (Figure 7a).

Future studies could investigate the hypothesized mechanisms for our current observations or investigate additional approaches to enhancing propagation of these thornforest species. For example, ethylene is another natural occurring hormone produced during rapid cell division and fruit ripening [41]. Ethylene is the only major plant hormone that occurs as a gas and is a very small molecule [42], so it may more easily penetrate the endocarp and reach the seed embryo in *C. boissieri*. *Cordia boissieri* fruits are relatively large fleshy drupes that often fall from the parent tree shortly after ripening. The ripening process releases ethylene, which can induce seed maturation and fruit ripening in nearby immature fruits [42]. Collection of fruits from trees prior to full maturation may explain the low germination rates frequently observed for *C. boissieri*, but this could potentially be countered with ethylene treatment, or even just storage practices that promote ethylene accumulation around harvested seeds or fruits.

Fewer comparisons with prior studies are possible for *C. boissieri* because soil mixture types and the effects of chemical seed treatments on seedling survival and performance have not previously been tested for *C. boissieri*. Unlike *E. ebano*, soil mixture treatments had only an interactive effect on *C. boissieri* emergence likelihood (pretreatment × soil, discussed above) (Table 9), and no effect on time to emergence or on seedling survival, height, or leaf abundance (Table 10, Table 11, Table 12 and Table 13). Nevertheless, soil B appears to have exhibited the highest emergence (23.3%) but the lowest seedling growth (16.5 mm height, 2.5 leaves), whereas soils C and F had the highest seedling growth (33.8 and 31.4 mm height, 4.8 and 5.8 leaves, respectively) but moderate emergence (16.7% and 15.0%), and soil C had the lowest seedling survival (80%). The effects of different soil types on *C. boissieri* germination and seedling performance merit additional study and, given the large proportion of *C. boissieri* seeds that experience mammalian gut passage, so do the nature and impacts of certain soil microbial associations.

Stratification also had fewer effects on germination and performance for *C. boissieri* than it did for *E. ebano*. However, like *E. ebano*, the effects stratification had were weakly negative, and there is no justification for either including stratification treatments in propagation protocols or avoiding cold storage for the sake of seed banking. As aforementioned, this finding contradicts a prior study on *C. boissieri* germination by Schuch et al. [40], who found warm stratification significantly increased germination. Schuch et al. [40] also found that *C. boissieri* seed longevity was relatively limited and called for further study of different storage methods, which we agree merit investigation.

*Zanthoxylum fagara* propagation remains a major challenge and an unresolved mystery. *Zanthoxylum fagara* germination was effectively zero, just as in a prior study using similar methods [15]. The likely causes for this paucity of germination were discussed at length previously [15], and the same factors likely applied in this study. Most importantly, we suspect that the low germination rates in this study were due to near-zero viability of the seeds tested. Unfortunately, this means we cannot even conclude that the current treatments were ineffective or unimportant, as they could have had strong effects on seed with higher viability. Use of fresh seed is now standard procedure for *Z. fagara* propagation at the regional USFWS nursery, but *Z. fagara* germination remains low and highly variable [35]. The current suite of factors tested remain worth investigating in the future, but perhaps more urgent are tests of *Z. fagara* seed longevity and evaluations of different seed harvesting and processing methods.

*Ebenopsis ebano*, *C. boissieri*, and *Z. fagara* are only three of the 75+ plant species that regularly make up Tamaulipan thornscrub forest communities, and many common horticulture techniques that could increase their propagation remain untested. Filling these knowledge gaps and developing a quantitative foundation to provide better, evidence-based propagation guidelines for thornforest species can directly and immediately increase not only seedling production, but also restored acreage and, by extension, regional biodiversity and other ecosystem services. Urbanization and human land use change continue to increase in the Lower Rio Grande Valley as its human population continues to grow rapidly. Now, more than ever, major advances in thornforest habitat restoration are needed if we are to return Tamaulipan thornforests to a regular part of the regional landscape, rather than a few scattered jewels with enemies at their gates.

## 4. Materials and Methods

Following logically from our previous study on (a) the individual effects of different levels of scarification and two phytohormone treatments (seed pretreatments) on germination and (b) the effects of soil type and soil warming on seedling performance of three focal thornforest species [15], our current approach was to examine the combined effects of scarification, phytohormones, soil type, and stratification on both germination and seedling growth. This study included two experiments for each focal species. The first was a smaller factorial experiment investigating the effects of combinations of different chemical seed treatments on germination. The second was a larger factorial experiment investigating the combined effects of chemical seed treatments, stratification treatments, and soil mixture type on both germination and, for *E. ebano*, seedling performance.

### 4.1. Study Site

The seed treatments and the smaller factorial experiments were performed in a laboratory at the University of Texas Rio Grande Valley (UTRGV) in Brownville, Texas, USA. The larger factorial experiments were performed outdoors at the Brownsville Research and Community Garden (BRCG) located on the same UTRGV campus in Brownville (25°53′44.5″ N, 97°28′54.3″ W) from 21 January 2020 to 30 May 2020. During this period, based on weather data collected at the Brownsville-South Padre Island International Airport (station ID USW00012919) located 6.1 km from the study site, the average temperature was 24.4 °C, and average daily high and low temperatures were 30.0 °C and 20.1 °C, respectively. Temperatures reached as high as 39.4 °C and as low as 4.4 °C. Rainfall in the same period was 5.4 cm and there was no snowfall. Rainfall was supplemented with regular manual watering every 2 days using a standard hose with shower nozzle attachment utilizing municipal water. Average wind speed was 11.40 km/h with a maximum 2-min wind speed of 23 km/h and a maximum 5-s wind speed of 29.4 km/h. No shade or other environmental manipulations were imposed on the experimental seedlings.

Seeds were wild collected at various locations within Cameron and Hidalgo Counties in the summer and fall of 2019. *Ebenopsis ebano* and *Z. fagara* seeds were primarily collected, with permission, from private residential properties, whereas *C. boissieri* seeds were primarily collected, with permission, from the UTRGV Brownsville and Edinburg campuses. Care was taken to ensure seeds were only collected from trees known to have recruited in place naturally. All trees were located within the geographic range required for federal thornforest restoration projects [16]. The protocols utilized for processing seeds prior to experimental treatments are described in Luera et al. [15]. Seeds of the same species from different source trees were combined and thoroughly mixed prior to treatments and experimentation. All seeds were stored at room temperature (20–22 °C) in a laboratory on the UTRGV Brownsville campus prior to experimental treatments.

Detailed descriptions of the three focal species may be found in Luera et al. [15].

### 4.2. Chemical Seed Treatment Combination Experiments

Seeds of each focal species were subjected to three chemical treatments in a factorial design using either the optimal dose identified by Luera et al. [15] (described below) or a corresponding control for sulfuric acid (SA), gibberellic acid 3 (GA), and indole-3-butyric acid (IBA). Thus, this experiment employed a full factorial design with 8 total treatment combinations: SA, GA, IBA, SA + GA, SA + IBA, GA + IBA, SA + GA + IBA, and control. SA treatments were always performed first, followed by GA treatments, and then IBA treatments. This order was necessary because the scarifying effect of SA would denature both GA and IBA, and because IBA was applied as a powder coating that would have been washed away during the GA solution soak.

All seed pretreatments were performed from 15–17 January 2021. All seeds were then placed into 100 mm × 15 mm petri dishes on precut moistened paper towels on 17 January 2021 and monitored daily for germination or molding until 20 March 2021. Each dish received either 12 *C. boissieri* seeds, 20 *E. ebano* seeds, or 20 *Z. fagara* seeds from a single treatment combination. Seeds were considered germinated once radical emergence was apparent and distinct. Molded seeds were gently squeezed to assess embryo death and discarded if dead. The surface sterilization protocol described in Luera et al. [15] was performed weekly.

Optimal dosages for the different chemical treatments were determined based on preliminary results from the Luera et al. [15] study. Thus, soaking times in 95% sulfuric acid (SA) (MilliporeSigma, Burlington, MA, USA) were 120 min for *C. boissieri*, 50 min for *E. ebano*, and 2 min for *Z. fagara*. Seeds subject to the SA control treatment were soaked in water for the designated time interval. Seeds subject to SA treatment were coated with SA and left undisturbed (without stirring) for the designated time before being neutralized in an agricultural lime bath, washed, and dried, as described in Luera et al. [15]. Optimal gibberellic acid (GA) dosages entailed soaking for 24 h in 100 mg/L aqueous gibberellic acid (gibberellin A3) solution (GoldBio, St. Louis, MO, USA) for both *C. boissieri* and *E. ebano*, and the same dosage was used for *Z. fagara* even though it did not respond to GA previously [15]. Seeds were soaked in GA solution at room temperatures for 24 h and stirred twice during that period before being drained and dried. Seeds subject to the control IBA treatment were then placed into petri dishes without any additional manipulation, whereas seeds subject to the optimal IBA treatment were coated with 3% (by mass) powdered indole-3-butyric acid (Hormex #30; Maia Products, Inc., Westlake Village, CA, USA) using the protocol described in Luera et al. [15] before being placed in petri dishes.

### 4.3. Chemical Pretreatment, Stratification, and Soil Type Experiments

For the second, larger factorial experiment, seeds were subjected to a subset of five of the prior chemical pretreatments (GA, IBA, SA, GA + IBA + SA, and control); three stratification treatments (cold, ambient control, or warm); and six soil mixture type treatments (described below). The chemical pretreatments were imposed using the same protocols described above. Prior to chemical pretreatment, stratification treatments were imposed by placing dry seeds into paper envelopes (to mimic the dry conditions the focal species typically experience in their arid native habitats) and subjecting them to one of three temperature regimes for 30 days: ambient control (room temperatures of ca. 21–23 °C), cold (ca. 4 °C in a refrigerator), or warm (ca. 38 °C in a drying oven). Stratifying seeds were observed weekly for notable changes and stored at room temperature for 7 days after temperature manipulation prior to chemical treatments. After seeds received their designated stratification and chemical treatments, one seed from each treatment combination was sown into a biodegradable paper container (3.8 cm wide × 3.8 cm long x 20.3 cm tall) filled with one the six designated soil mixture types.

Soils were mixed by volume as recommended by Wahl-Villareal [16] using the protocols described by Luera et al. [15]. Slow release Osmocote Pro 19-5-9 granular fertilizer (ICL Fertilizers, Dublin, OH) were utilized at the recommended rates [16] and mixed until homogenized. The proportions of different soil media components in the experimental soil mixture types were as follows: (A) 50% vermiculite, 50% topsoil; (B) 50% topsoil, 25% perlite, 25% vermiculite; (C) 50% peat moss, 25% sand, 25% topsoil; (D) 50% peat moss, 25% sand, 25% vermiculite; (E) 50% peat moss, 25% sand, 25% perlite; and (F) 50% peat moss, 20% vermiculite, 20% topsoil, 10% perlite. Tap water was added to soil mixtures 24 h prior to planting to allow peat moss and other substrates to become hydrated. Planted containers were thoroughly watered immediately after planting to ensure all soil mixtures began at field capacity, and all containers were then maintained outdoors in full sun at the UTRGV Brownsville campus.

Each of the 90 treatment combinations (5 × 3 × 6 full factorial design) was replicated four times, yielding 360 total seeds for each of the three focal species and with each seed in an independent container. We could not assess germination of seeds sown in soil by observing radicle emergence, as we did for the smaller factorial experiment, so we assessed germination based on whether and when seedlings successfully emerged from the soil surface. Germination in the smaller experiments and emergence in the larger experiments were recorded daily.

### 4.4. Statistical Analyses

For binary response variables (seed germination or emergence and seedling survival), we fit generalized linear models (GLMs) for each species using the ‘glm’ function in R version 4.1.0 (R Foundation for Statistical Computing, Vienna, Austria) with a binomial distribution family and model terms for applicable treatments and interactions. For each GLM, we performed an analysis of deviance (ANODEV) to examine differences among experimental treatments, followed by least squares means post-hoc tests (‘lsmeans’ function in R), where applicable, to identify significant differences between treatment levels. For continuous response variables (time to germination, seedling height, and seedling leaf count), we fit linear models using the ‘lm’ function in R with model terms for applicable treatments. We then used analysis of variance (ANOVA), or, if a model included seedling age, analysis of covariance (ANCOVA) to evaluate the effects of experimental treatments with least squares means post-hoc tests to compare treatment levels.

We performed Shapiro–Wilk tests of normality (‘shapiro.test’ function in R) on model residuals and Breusch–Pagan tests for homoscedasticity (‘bptest’ function in R) for each linear model to assess whether our models met the assumptions of ANOVA. We calculated variance inflation factors (‘vif’ function in R) for all models to confirm they did not violate assumptions of multicollinearity. Accordingly, we square root transformed the time to germination for *E. ebano* in the small factorial experiment to meet the assumptions of ANOVA. However, emergence time and height for the *E. ebano* large factorial experiment and height and leaf count for the *C. boissieri* large factorial experiment could not be transformed to achieve normality, nor did the observations for these variables match any probability distribution functions that would permit successful modeling using GLMs, so we fit permutational linear models for these variables using the ‘lmp’ function in R. This function used a non-parametric randomization procedure with 10,000 iterations to generate bootstrapped F and *p* values.

The full models for days to germination in the *E. ebano* small factorial experiment and in both *C. boissieri* experiments had low statistical power, so we performed stepwise model pruning using the ‘step’ function in R to increase our statistical power by removing terms that were not significant and explained the least observed variance. We used Tukey adjustments in our least squares means post-hoc tests when comparing more than 12 treatment levels, otherwise our post-hoc tests were unadjusted. A probability value of *p* < 0.05 was used to determine significance.

## 5. Conclusions

Sulfuric acid (SA) scarification was required for *E. ebano* germination and was the only chemical pretreatment to influence germination likelihood, even in combination with SA. This study does not support the suggestion by Luera et al. [15] that treating *E. ebano* seeds with phytohormones after scarification might further enhance germination. Seed treatments with gibberellic acid (GA) and indole-3-butyric acid (IBA) reduced *E. ebano* time to germination, but only by ca. 2–3 days, which provides little benefit and would not justify the cost or effort in most circumstances. Cold and warm stratification reduced germination compared to controls and should be avoided, but cold storage is still a viable long-term storage option. Soil type was important but came with tradeoffs. After accounting for all other factors (residual analysis), *E. ebano* germination was ca. 15–40% higher in soil type D (50% peat moss, 25% sand, 25% vermiculite) than in other soil types and lowest in types C and E; however, soil C (50% peat moss, 25% sand, 25% topsoil) produced some of the tallest and most foliose seedlings. Seedling survival was ca. 10–17% higher in soils C, D, and F compared to others. We are reluctant, however, to unreservedly promote use of substrates high in peat moss for four reasons. First, Luera et al. [15] documented many benefits among soil mixtures higher in native topsoil, especially under adverse conditions and warming scenarios. Second, practitioners have observed elevated mortality rates immediately following field transplantation among seedlings grown in peat moss-based substrates [43]. Third is the cost of peat moss relative to topsoil. Fourth, peat moss raises more concerns about environmental impacts and sustainability than local topsoil, though this depends on how these substrates are sourced, harvested, and processed.

*Cordia boissieri* seeds germinated best with GA treatment and benefitted from combining SA and IBA treatments with GA in a realistic nursery context. IBA reduced germination of *C. boissieri* germination in vitro, but likely did so by promoting mold growth in ways not likely to occur in a nursery context. We currently recommend treating *C. boissieri* seeds with GA + IBA + SA and using an aqueous IBA solution rather than its common powder form. However, additional nursery trials comparing GA + IBA, GA + SA, and GA + IBA + SA are merited. Seed pretreatments had weak marginal effects on plant height. Seedlings treated with GA only were slightly taller than GA + IBA + SA treatments, but survival was equivalent and the increase in germination seen with GA + IBA + SA would be more important than this difference in height in most contexts.

*Zanthoxylum fagara* germination was near zero across all treatments in both experiments; thus, *Z. fagara* propagation remains poorly understood and a challenge for practitioners. Current best practices are to use fresh seed in order to maximize viability [35,43]. The factors tested in this study remain worth investigating, but studies of *Z. fagara* seed longevity and comparisons of different seed harvesting and processing methods are more urgent and likely to provide greater immediate benefits.

## Figures and Tables

**Table 1:** ANODEV results examining the effects of sulfuric acid, gibberellic acid, indole-3-butyric acid, and all interactions thereof on the germination likelihood of *Ebenopsis ebano* seeds.

Factor	d.f.	χ^2^	*p*	
Sulfuric acid (SA)	1	129.73	<0.0001	***
Gibberellic acid (GA)	1	0.45	0.5014	
Indole-3-butyric acid (IBA)	1	0.05	0.8224	
SA × GA	1	1.16	0.2823	
SA × IBA	1	1.36	0.2440	
GA × IBA	1	0.00	1.0000	
SA × GA × IBA	1	0.00	1.0000	
Model	11	106.92	<0.0001	***

Legend: d.f., degrees of freedom; χ^2^, chi-squared test statistic; *p*, *p*-value; ***, *p* < 0.001.

**Table 2:** ANOVA results examining the effects of sulfuric acid, gibberellic acid, indole-3-butyric acid, and the GA × IBA interaction on time to germination of *Ebenopsis ebano* seeds.

Factor	d.f.	*F*_4,66_	*p*	
Sulfuric acid (SA)	1	0.01	0.9287	
Gibberellic acid (GA)	1	0.58	0.4507	
Indole 3-butyric acid (IBA)	1	4.84	0.0312	*
GA × IBA	1	5.03	0.0289	*
Model	4	2.61	0.0430	*

Legend: d.f., degrees of freedom; *F*_4,66_, *F* test statistic; *p*, *p*-value; *, *p* < 0.05.

**Table 3:** ANODEV results examining the effects of chemical seed pretreatment, soil mixture type, stratification, and all interactions thereof on *Ebenopsis ebano* emergence likelihood.

Factor	d.f.	χ^2^	*p*	
Pretreatment	4	261.76	<0.0001	***
Soil type	5	13.12	0.0223	*
Stratification	2	5.92	0.0517	.
Pretreatment × Soil	20	28.29	0.1026	
Pretreatment × Strat	8	15.82	0.0449	*
Soil × Strat	10	9.92	0.4471	
Pretreatment × Soil × Strat	40	12.97	0.9999	
Model	89	347.82	<0.0001	***

Legend: d.f., degrees of freedom; χ^2^, chi-squared test statistic; *p*, *p*-value; ., 0.1 > *p* ≥ 0. 05; *, 0.05 > *p* ≥ 0.01; ***, *p* < 0.001.

**Table 4:** Permutational ANOVA results examining the effects of chemical seed pretreatment, soil mixture type, stratification, and all interactions thereof on time to *Ebenopsis ebano* seedling emergence. Pretreatments that lacked SA were excluded from analysis.

Factor	d.f.	*F*_35,83_	*p*	
Pretreatment	1	4.88	0.0299	*
Soil type	5	1.85	0.1123	
Stratification	2	1.49	0.2316	
Pretreatment × Soil	5	7.44	<0.0001	***
Pretreatment × Strat	2	13.66	<0.0001	***
Soil × Strat	10	2.22	0.0243	*
Pretreatment × Soil × Strat	10	0.72	0.7009	
Model	35	6.20	<0.0001	***

Legend: d.f., degrees of freedom; *F*_35,83_, *F* test statistic; *p*, *p*-value; *, 0.05 > *p* ≥ 0.01; ***, *p* < 0.001.

**Table 5:** ANODEV results examining the effects of chemical seed pretreatment, soil mixture type, stratification, and all second-order interactions on *Ebenopsis ebano* seedling survival. Pretreatments that lacked SA were excluded from analysis.

Factor	d.f.	*F*_35,83_	*p*	
Pretreatment	1	1.45	0.2289	
Soil type	5	9.96	0.0764	.
Stratification	2	1.72	0.4238	
Pretreatment × Soil	5	0.94	0.9670	
Pretreatment × Strat	2	2.00	0.3687	
Soil × Strat	10	9.83	0.4558	
Model	25	34.53	0.4420	

Legend: d.f., degrees of freedom; *F*_35,83_, *F* test statistic; *p*, *p*-value; ., 0.1 > *p* ≥ 0.05.

**Table 6:** Permutational ANCOVA results examining the effects of seedling age, chemical pretreatment, soil mixture type, stratification, and all second-order interactions on *Ebenopsis ebano* seedling height. Pretreatments that lacked SA were excluded from analysis.

Factor	d.f.	*F*_34,74_	*p*	
Seedling age	1	10.68	<0.0001	***
Pretreatment	1	1.21	0.1615	
Soil type	5	1.38	1.0000	
Stratification	2	2.20	0.4024	
Age × Pretreat	1	1.12	0.3004	
Age × Soil	5	1.42	0.2554	
Age × Strat	2	2.49	0.0877	.
Pretreatment × Soil	5	3.51	0.0172	*
Pretreatment × Strat	2	0.01	1.0000	
Soil × Strat	10	1.93	0.0644	.
Model	34	1.96	0.0083	**

Legend: d.f., degrees of freedom; *F*_34,74_, *F* test statistic; *p*, *p*-value; ., 0.1 > *p* ≥ 0.05; *, 0.05 > *p* ≥ 0.01; **, 0.01 > *p* ≥ 0.001; ***, *p* < 0.001.

**Table 7:** ANCOVA results examining the effects of seedling age, chemical pretreatment, soil mixture type, stratification, and the interactions of pretreatment × soil, pretreatment × stratification, soil × stratification, and pretreatment × soil × stratification on *Ebenopsis ebano* seedling leaf abundance. We initially included interaction terms with seedling age but pruned the model to increase statistical power. Pretreatments that lacked SA were excluded from analysis.

Factor	d.f.	*F*_36,72_	*p*	
Seedling age	1	13.80	0.0004	***
Pretreatment	1	0.017	0.8983	
Soil type	5	2.35	0.0492	*
Stratification	2	0.23	0.7927	
Pretreatment × Soil	5	0.37	0.8649	
Pretreatment × Strat	2	0.57	0.5663	
Soil × Strat	10	1.56	0.1353	
Pretreatment × Soil × Strat	10	1.60	0.1228	
Model	36	1.98	0.0070	**

Legend: d.f., degrees of freedom; *F*_36,72_, *F* test statistic; *p*, *p*-value; *, 0.05 > *p* ≥ 0.01; **, 0.01 > *p* ≥ 0.001; ***, *p* < 0.001.

**Table 8:** ANODEV results examining the effects of sulfuric acid, gibberellic acid, indole-3-butyric acid, and all interactions thereof on the germination likelihood of *Cordia boissieri* seeds.

Factor	d.f.	χ^2^	*p*	
Sulfuric acid (SA)	1	0.16	0.6908	
Gibberellic acid (GA)	1	17.04	<0.0001	***
Indole-3-butyric acid (IBA)	1	8.73	0.0031	**
SA × GA	1	0.56	0.4551	
SA × IBA	1	14.17	0.0002	***
GA × IBA	1	3.26	0.0710	.
SA × GA × IBA	1	0.00	1.0000	
Model	7	43.92	<0.0001	***

Legend: d.f., degrees of freedom; χ^2^, chi-squared test statistic; *p*, *p*-value; ., 0.1 > *p* ≥ 0.05; **, 0.01 > *p* ≥ 0.001; ***, *p* < 0.001.

**Table 9:** ANODEV results examining the effects of chemical pretreatment, soil mixture type, stratification, and all interactions thereof on *Cordia boissieri* emergence likelihood.

Factor	d.f.	χ^2^	*p*	
Pretreatment	4	80.99	<0.0001	***
Soil type	5	3.27	0.6589	
Stratification	2	2.81	0.2451	
Pretreatment × Soil	20	31.62	0.0476	*
Pretreatment × Strat	8	11.19	0.1913	
Soil × Strat	10	13.05	0.2208	
Pretreatment × Soil × Strat	40	25.76	0.9605	
Model	89	168.68	<0.0001	***

Legend: d.f., degrees of freedom; χ^2^, chi-squared statistic; *p*, *p*-value; *, 0.05 > *p* ≥ 0.01; ***, *p* < 0.001.

**Table 10:** ANOVA results examining the effects of chemical pretreatment, soil mixture type, and stratification on *Cordia boissieri* time to emergence. Interaction terms were initially considered but pruned from the model to increase statistical power.

Factor	d.f.	*F*_11,47_	*p*	
Pretreatment	4	4.28	0.0049	**
Soil type	5	1.71	0.1510	
Stratification	2	1.07	0.3511	
Model	11	2.53	0.0136	*

Legend: d.f., degrees of freedom; *F*_11,47_, *F* test statistic; *p*, *p*-value; *, 0.05 > *p* ≥ 0.01; **, 0.01 > *p* ≥ 0.001.

**Table 11:** ANODEV results examining the effects of chemical pretreatment, soil mixture type, and stratification on *Cordia boissieri* seedling survival. Interaction terms were initially considered but pruned from the model to increase statistical power.

Factor	d.f.	χ^2^	*p*
Pretreatment	4	26.12	0.4922
Soil type	5	19.66	0.2642
Stratification	2	19.23	0.8056
Model	11	10.30	0.5039

Legend: d.f., degrees of freedom; χ^2^, chi-squared test statistic; *p*, *p*-value.

**Table 12:** Permutational ANCOVA results examining the effects of seedling age, chemical seed pretreatment, soil mixture type, stratification, and the interactions of pretreatment × soil type, pretreatment × stratification, and soil type × stratification on *Cordia boissieri* seedling height. Additional interaction terms were initially considered but pruned from the model to increase statistical power.

Factor	d.f.	*F*_31,21_	*p*	
Seedling age	1	12.74	0.0031	**
Pretreatment	4	2.95	0.0685	.
Soil type	5	1.35	0.2687	
Stratification	2	5.16	0.0300	*
Pretreatment × Soil	6	2.25	0.1013	
Pretreatment × Strat	4	1.92	0.1932	
Soil × Strat	9	1.90	0.1756	
Model	31	2.69	0.0104	*

Legend: d.f., degrees of freedom; *F*_31,21_, *F* test statistic; *p*, *p*-value; ., 0.1 > *p* ≥ 0.05; *, 0.05 > *p* ≥ 0.01; **, 0.01 > *p* ≥ 0.001.

**Table 13:** Permutational ANCOVA results examining the effects of seedling age, chemical seed pretreatment, soil mixture type, stratification, and the interactions of pretreatment × soil type, pretreatment × stratification, and soil type × stratification on *Cordia boissieri* seedling leaf abundance. Additional interaction terms were initially considered but pruned from the model to increase statistical power.

Factor	d.f.	*F*_31,21_	*p*	
Seedling age	1	17.94	0.0013	**
Pretreatment	4	0.48	0.6729	
Soil type	5	0.37	0.7404	
Stratification	2	0.48	0.9608	
Pretreatment × Soil	6	0.35	0.8013	
Pretreatment × Strat	4	1.09	0.4620	
Soil × Strat	9	0.43	0.9146	
Model	31	2.42	0.0191	*

Legend: d.f., degrees of freedom; *F*_31,21_, *F* test statistic; *p*, *p*-value; *, 0.05 > *p* ≥ 0.01; **, 0.01 > *p* ≥ 0.001.

## Data Availability

The data generated by this experiment and used for the analyses reported herein are available upon request from the corresponding author.
